# Integrating Perceived Psychosocial Outcomes Into the Assessment of Telecare Acceptability Among Slovenian Informal Caregivers of Older People: Cross-Sectional Survey Study

**DOI:** 10.2196/85534

**Published:** 2026-10-20

**Authors:** Vesna Dolničar, Nejc Berzelak, Sakari Taipale, Simona Hvalič-Touzery

**Affiliations:** 1 Faculty of Social Sciences, University of Ljubljana, Kardeljeva pl. 5, Ljubljana, 1000, Slovenia, 386 1-5805-366; 2 Social Protection Institute of the Republic of Slovenia, Ljubljana, Slovenia; 3 Department of Social Sciences and Philosophy, University of Jyväskylä, Jyväskylä, Finland

**Keywords:** aging, behavioral intention to use, e-care, informal carers, informal care, older adults, psychosocial factors, psychosocial impacts, technology acceptance, telecare, Unified Theory of Acceptance and Use of Technology, UTAUT

## Abstract

**Background:**

While telecare has considerable potential to improve home care services, benefit older adults, and support informal caregivers, its adoption remains limited. There is also a notable gap in empirical research on the factors that influence informal caregivers’ behavioral intention to use telecare. In particular, existing theoretical acceptance models are insufficiently comprehensive, as they do not specifically address informal caregivers of older adults and have largely overlooked the psychosocial outcomes of engaging with telecare.

**Objective:**

This study examined the behavioral intention of Slovenian informal caregivers of older adults to use telecare, focusing on the preuse stage of technology acceptance, by integrating the perceived psychosocial outcomes of telecare engagement (peace of mind, reduced anxiety, sense of safety, and perceived connectedness with the care recipient) into the Unified Theory of Acceptance and Use of Technology (UTAUT) model.

**Methods:**

The study applied nested ordinary least squares regression analysis to survey data collected in 2021 in Slovenia from informal caregivers of older adults, all of whom were aged 40 years or older. Of 612 respondents in the dataset, 558 were retained after excluding current or former users of care-related assistive technologies; the regression analyses were based on the 526 participants with complete data on all model variables.

**Results:**

The regression analysis used 3 nested models to examine predictors of the intention of informal caregivers of older adults to use telecare. The first model, which included only background variables (burden of care, financial coping, and caregiver sociodemographic characteristics), had limited explanatory power (*R*²=0.03), with financial coping as the only significant predictor (β=−0.14, *P*=.005). Adding UTAUT predictors significantly improved the model’s fit (*R*²=0.49). Performance expectancy (β=0.52, *P*<.001), facilitating conditions (β=0.11, *P*=.005), and social influence (β=0.21, *P*<.001) emerged as additional significant predictors. Incorporating perceived psychosocial outcomes further improved the model’s fit and increased the explained variance (*R*²=0.54); this additional factor also showed the strongest association (β=0.41, *P*<.001). Patterns for the other predictors remained consistent across models, but including perceived psychosocial outcomes substantially reduced the coefficient for performance expectancy.

**Conclusions:**

In this cross-sectional sample, perceived psychosocial outcomes were the predictors most strongly associated with behavioral intention in the extended UTAUT model, indicating that they may be important factors in understanding telecare acceptability among informal caregivers. The findings suggest that developers, policymakers, and health and social care providers should consider not only functional aspects of telecare but also the psychosocial needs of informal caregivers. As the study measured self-reported intention at the preuse stage rather than actual use, longitudinal and behavioral research is needed to establish whether these associations translate into telecare adoption.

## Introduction

### Background

Population aging and the increasing prevalence of multiple chronic conditions among older adults are amplifying demand for caregiving services [1,2]. In this context, informal caregivers, who provide unpaid care to someone with a long-term illness, disability, or other long-lasting health or care need outside professional or formal frameworks, are essential [3]. Globally, the number of individuals providing such care is increasing [4,5], with informal caregivers representing a substantial share of the population in many countries, such as 9% in the United Kingdom and 11.5% in the United States [6-8]. In Europe, they provide 80% of all care [3,9,10], making them the largest providers of social care. In Slovenia, informal caregivers form the backbone of the long-term care system [11], with 23.6% of the adult population providing informal care to older adults [12]. This reflects a familialist care model in which the caring responsibilities for older people primarily fall on the family [13,14]. Despite this reliance, strong policies to support and facilitate these responsibilities are lacking [3,11,14].

Caregiving responsibilities can be extensive and demanding. They often place a significant burden on informal caregivers and affect their physical and emotional well-being [11,15-18]. They are likely to experience significant stress when they lack the internal and external resources needed to manage their caregiving responsibilities [19].

In this context, there is growing evidence supporting the potential benefits of telecare solutions in empowering and supporting informal caregivers [9,10,20-24]. In informal care for older adults, the term telecare refers to services that use digital technologies to enable older people to live more independently and safely in their homes for longer [25,26]. Telecare encompasses many services and devices that use digital technology to provide remote health care and social support, including personal alarm systems, trackers, environmental sensors, and reminders. Such services can also help informal caregivers manage their caregiving responsibilities more efficiently while offering peace of mind by providing reassurance about the well-being of the person they care for [25,26]. These technologies can thus represent supportive external resources that can relieve some of the pressure on informal caregivers.

While telecare has considerable promise for improving home care services, benefiting older people, and helping informal caregivers, its adoption remains limited [20,23,26]. Nevertheless, according to a report by Berg Insight [27], the number of telecare users in Europe was 9.0 million at the end of 2023 and is expected to exceed 13.1 million by 2028. This growth, fueled by an aging population and the increasing prevalence of chronic diseases [1,2], indicates the scale of potential telecare use. Notwithstanding the growing need and potential demand, several challenges hinder the wider implementation of telecare solutions [9,28,29]. In particular, their acceptability to informal caregivers is a key barrier [16,25,26,30].

### Theoretical Framework and Goal of the Study

Although telecare offers significant potential in informal caregiving, there is a lack of research into the factors that influence informal caregivers’ behavioral intention to use it [23,26]. Existing research in this area typically focuses on the classic variables of technology acceptance models: usefulness and ease of use. Several theoretical models have been applied to explore the factors influencing the intention to use telecare [31-34], including the health belief model [35], theory of reasoned action [36], theory of planned behavior [36], diffusion of innovations theory [37], domestication theory [38], technology acceptance model [39], and Unified Theory of Acceptance and Use of Technology (UTAUT) [40]. Among these, UTAUT was selected as the theoretical framework for this study because it integrates key determinants from earlier technology acceptance models and has been widely applied in studies examining the acceptance of digital health and assistive technologies, including research involving informal caregivers [32,41].

While UTAUT provides a solid foundation for examining factors influencing the intention to use telecare, it was not specifically developed to explain telecare use among informal caregivers of older adults and therefore does not adequately capture factors particularly relevant in health care settings [42] and informal caregiving contexts [43]. Furthermore, it and other telecare acceptance models have largely overlooked the psychosocial outcomes [23,43] associated with telecare engagement, such as peace of mind, reduced anxiety, and a sense of safety. These outcomes are particularly relevant in informal caregiving contexts because telecare technologies are often valued not only for functional support but also for the emotional reassurance they provide to caregivers [23,44-47]. Psychosocial outcomes of telecare engagement refer to the mediating effects of both internal factors (such as psychological and emotional responses) and external sociotechnical environments on an individual’s social and psychological well-being [48-50]. In this context, telecare is part of the sociotechnical environment that shapes informal caregivers’ responses while delivering unpaid care to older adults within a particular social and structural framework. The psychosocial outcomes of telecare engagement can then affect psychological and social functioning, feelings, behaviors, relationships, interactions, and cognitive processes [51]. A recent scoping study [23] identified peace of mind, reduced anxiety, a sense of safety, and perceived connectedness with the care recipient as the most common psychosocial outcomes of telecare engagement among informal caregivers.

To address the limitations of UTAUT and other acceptance models in this field and to enhance understanding of telecare acceptability among informal caregivers of older people, this study aims to integrate the perceived psychosocial outcomes of telecare engagement into the UTAUT model. The dependent variable in the UTAUT model is the behavioral intention to use telecare, defined as a measure of the strength of one’s intention to perform a specified behavior [40,52-54]. Behavioral intention refers to a person’s motivation to engage in a specific behavior [52]; the more a person is willing to perform a particular behavior, the more likely it is that the behavior will be performed [53,54].

The aim of this study is, therefore, to examine the behavioral intention of Slovenian informal caregivers of older adults to use telecare by integrating perceived psychosocial outcomes into the UTAUT model. According to the UTAUT model, behavioral intention is influenced by performance expectancy (the degree to which using the system is perceived to improve performance), effort expectancy (the degree of ease associated with system use), social influence (the degree to which important others are perceived to influence its use), and facilitating conditions (the degree to which resources and support are perceived to be available) [40]. We extend this model by including perceived psychosocial outcomes of telecare engagement as an additional predictor (Figure 1). Based on prior research [23,25,55-57], we hypothesize that perceived psychosocial outcomes will positively predict the behavioral intention to use telecare.

**Figure 1.**
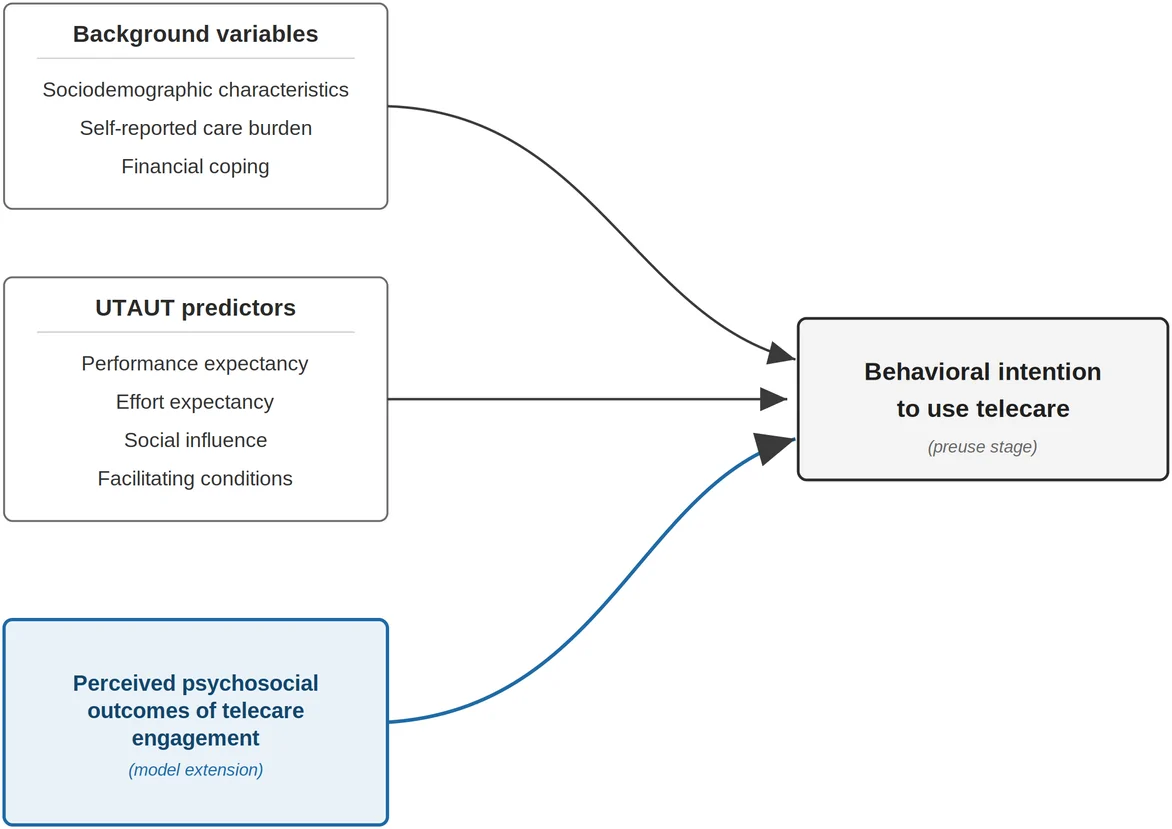
Conceptual model of telecare acceptability among informal caregivers, extending the Unified Theory of Acceptance and Use of Technology (UTAUT) framework with perceived psychosocial outcomes. The blue box represents the model extension. Perceived psychosocial outcomes comprise 4 indicators: peace of mind, reduced anxiety, sense of safety, and perceived connectedness with the care recipient.

This study thus addresses the following research question: how are background characteristics (sociodemographics, self-reported care burden, and financial coping), UTAUT constructs (performance expectancy, effort expectancy, social influence, and facilitating conditions), and the perceived psychosocial outcomes of telecare engagement associated with the behavioral intention of Slovenian informal caregivers of older people to use telecare? The study makes a theoretical, methodological, and practical contribution by extending the UTAUT model with psychosocial outcomes, providing empirical evidence on telecare acceptability among informal caregivers of older people in Slovenia, and offering insights relevant to the design and implementation of telecare solutions.

Following the technology acceptance lifecycle [43], which conceptualizes technology acceptance as a staged process, this study focuses on the initial preadoption stage (termed “preuse acceptability” [43]), which occurs prior to the actual first use of the technology, rather than on actual use or postadoption experiences. The research concentrates on 4 distinct telecare functionalities, grouped into 2 main categories: personal alarm systems, comprising the emergency call button and automatic fall detection, and mobility-related devices, comprising home activity sensors and location-tracking devices. Telecare acceptability is thus captured through behavioral intention to use, reflecting caregivers’ motivational readiness to use the technology in the preuse stage.

## Methods

### Study Design

The study population was Slovenian residents aged 40 years or older who had provided at least 4 hours of unpaid care per week to a family member or relative aged 65 years or over during the previous 6 months. The lower age threshold of 40 years was applied because international evidence indicates a higher prevalence of informal caregiving in this age group [58,59], while comparable population-level data on informal caregivers were not available for Slovenia. Unpaid care was defined as the provision of activities of daily living, instrumental activities of daily living, or emotional support.

In the absence of appropriate sampling frames for informal caregivers, a survey with a probability sample of the general population was not feasible. Estimates of the prevalence of informal caregivers vary substantially across studies, depending on the definitions and characteristics of care recipients, which further complicates the construction of a reliable sampling frame [59]. Considering estimates of the proportion of informal caregivers in Slovenia in the target age group [60,61], such an approach would require large-scale screening to identify eligible individuals, which would result in prohibitive costs and practical challenges. We therefore used a nonprobability online panel with web survey data collection as a feasible approach to accessing this hard-to-reach population. This approach is increasingly used in social research to survey relatively small and difficult-to-identify population segments. Because nonprobability online panels may vary considerably in quality [62], we paid particular attention to the choice of panel provider, including an assessment against established ESOMAR guidelines for online panels [63]. The study requirements were clearly laid out, and the contract with the selected provider included a minimum sample size, within-panel selection and recruitment guidelines, control over questionnaire content and format, and clear reporting of the sample recruitment and data collection procedures.

### Participants and Recruitment

Data were collected between April and May 2021. The panel provider sent invitations to individuals selected from the panel members by quota sampling. Quotas were defined by 10-year age groups, sex, and region to approximate the structure of the general population rather than that of the informal caregiver population due to the lack of reliable population-level data about informal caregivers. In addition, to improve the comparability of the sample with the population of informal caregivers, we incorporated screening questions into the representative Slovenian Public Opinion Survey [64] to identify family caregivers and obtain their sociodemographic characteristics. These data were used to calculate poststratification weights.

Eligible respondents were identified with a screening web questionnaire consisting of questions about the respondent’s age, types of care provided to a family member, and the average number of hours spent weekly on care provision. Respondents with answers matching the definition of the target population were immediately routed to the main web questionnaire. Respondents who completed the questionnaire were assigned value points according to the standard incentivization practice of the panel provider. Information about voluntary participation, survey length, and data protection measures was provided, and no personally identifiable information about the participants was obtained. The respondents were allowed to skip individual questions and change their answers until the questionnaire was completed. To reduce nonresponse bias, the panel provider sent at least one email reminder to nonrespondents before attempting to contact additional panelists to meet the preset quotas. Access to the survey was limited to the invited panelists via unique access codes, and a respondent was able to participate in the survey only once.

Of 3284 panelists invited, 1834 completed the screening questionnaire and 781 were eligible. After excluding invalid, duplicate, and low-quality entries and respondents with insufficient completion or ineligible answers, 612 (18.6%) respondents remained in the final dataset. As the study focused on preuse behavioral intentions rather than user experience, 54 respondents were excluded because they were current or former users of care-related assistive technologies, such as emergency call services, fall detectors, sensors, or mobility trackers, or had failed to answer questions on prior use. This distinction is important, as factors influencing preuse behavioral intentions may differ from those shaping actual usage experience. This group was relatively small, reflecting the limited use of telecare services in Slovenia at the time of the study. A total of 558 participants were therefore included in the analysis. In summary, 1834 of the 3284 invited panelists completed the screening questionnaire, 612 were retained after exclusions, and 558 remained after removing current or former users of care-related assistive technologies. This sample of 558 was used for the reliability and factor analyses, while the regression analyses were based on the 526 participants with complete data on all model variables (see *Data Quality and Missing Data* section).

### Instruments and Measures

#### Questionnaire

The main questionnaire had 64 questions, with several of these comprising multiple items (see Multimedia Appendix 1 for key items; full materials are available online [65]). The number of questions presented varied across respondents because inapplicable questions were automatically skipped based on relevant previous answers. The mean questionnaire completion time after screening was 17 minutes.

The questionnaire included items on participants’ care provision and personal characteristics, as well as the study constructs. Self-reported care burden was measured using the Self-Rated Burden Scale [66], a single-item self-assessment scale ranging from 0 (“not burdensome at all”) to 10 (“extremely burdensome”), with higher scores indicating greater burden. UTAUT constructs and behavioral intention were measured using items adapted from prior studies, while perceived psychosocial outcomes were assessed separately through items capturing peace of mind, reduced anxiety, sense of safety, and perceived connectedness with the care recipient. The questionnaire was developed by the research team and evaluated in a multistage process, including initial pilot testing with informal caregivers (n=93), cognitive interviews (n=9), expert review (n=4), and a final pilot test (n=49), to identify and address potential issues.

#### Measures of Behavioral Intention

In this study, behavioral intention represents a person’s motivational readiness to engage with telecare [40,52,53], that is, how firmly they plan or are willing to use it. We operationalized the construct as the willingness to invest personal resources, specifically, time and money, to reflect the commitment they are prepared to make to potentially use the technology. This operationalization reflects the typical cost of comparable telecare services in Slovenia and was adopted because pretesting indicated that respondents had difficulty assessing their intention to use such services without information on potential costs, particularly given the low levels of awareness and use of telecare at the time of data collection. Behavioral intention to use telecare was assessed as the average score of 2 items, adapted from the UTAUT behavioral intention items [40], and measured on 5-point agreement scales ranging from 1 (strongly disagree) to 5 (strongly agree): “I would be willing to devote some time to learn how to properly use the services” and “I would be willing to pay 25 EUR (€1=US $1.14 as of September 21, 2026) per month for the services.”

#### UTAUT Measures and Perceived Psychosocial Outcomes

Four types of perceived psychosocial outcomes of telecare engagement identified in a prior scoping study [23] were measured: peace of mind, reduced anxiety, sense of safety, and perceived connectedness with the care recipient. For the exact wording of the questionnaire items, and their assignment to individual constructs, see Multimedia Appendix 1. All items were rated on a 5-point Likert-type scale from 1 (strongly disagree) to 5 (strongly agree). Four UTAUT constructs (performance expectancy, facilitating conditions, effort expectancy, and social influence) were measured with items adapted from prior studies [39,40,67], using the same response scale. Scores were calculated as means of the responses to their respective items.

#### Measurement Characteristics

The Cronbach α values for the scales with 3 or more items indicated good-to-acceptable reliability, with α=0.87 for perceived psychosocial outcomes, α=0.88 for performance expectancy, and α=0.62 for facilitating conditions. The somewhat lower α for facilitating conditions can be partially attributed to the scale’s small item count (3 items). The mean interitem correlation (*r*=0.35) falls within the recommended range of 0.2 to 0.4 [68]; however, α is mathematically constrained by the number of items for a given level of interitem correlation [69]. Given this, the obtained alpha is generally considered acceptable in exploratory research [70].

A confirmatory factor analysis supported the expected one-factor structure of the measures for the 3 scales comprising 3 or more items. Standardized loadings were all significant (*P*<.001), ranging between 0.73 and 0.91 for perceived psychosocial outcomes, between 0.62 and 0.86 for performance expectancy, and between 0.52 and 0.70 for facilitating conditions. Model fit was evaluated using the root mean square error of approximation (RMSEA), comparative fit index (CFI), Tucker-Lewis index (TLI), and standardized root mean square residual (SRMR). Model fit was good for perceived psychosocial outcomes (*χ*²_1_=0.92, *P*=.34, RMSEA<0.001, CFI>0.99, TLI>0.99, SRMR=0.004) and for performance expectancy (*χ*²_3_=3.26, *P*=.35, RMSEA=0.013, CFI >0.99, TLI>0.99, SRMR=0.004). Because the factor model for facilitating conditions was just identified, fit indices could not be computed.

#### Common Method Bias

Given that the data were collected using a single self-report survey, we assessed the potential for common method bias using the Harman single-factor test. The first factor accounted for 41% of the variance, which is below the commonly accepted threshold of 50% [71]. These results indicate that a single factor did not account for the majority of the variance, suggesting that common method bias is unlikely to substantially affect the findings. However, the Harman single-factor test has recognized limitations and cannot conclusively rule out the presence of common method variance. To further mitigate this risk, several procedural remedies were applied, including the use of validated measurement scales, careful questionnaire design, comprehensive pretesting, and assurances of respondent confidentiality, thereby reducing the likelihood of systematic response bias and common method bias.

### Data Analysis

#### Methods of Analysis

Univariate distributions of all the included variables were inspected before commencing the data analysis. The measurement properties of the latent constructs were evaluated using correlational analyses, reliability (internal consistency) analyses, and exploratory and confirmatory factor analyses.

We used nested ordinary least squares regression to determine the degree to which adding perceived psychosocial outcomes to the UTAUT model increases its explanatory power. Behavioral intention to use telecare was specified as the dependent variable, while background characteristics, UTAUT constructs, and perceived psychosocial outcomes were treated as independent variables. Since the dependent variable was constructed as the average of two 5-point agreement scale items, the resulting composite measure was treated as approximately continuous, following common practice in the literature. Accordingly, ordinary least squares regression was considered appropriate for the analysis, and the results were robust to alternative specifications using ordered logit models.

First, we fit a model containing only background variables (burden of care and the sociodemographic characteristics of caregivers) as predictors. Second, we added the scores for the 4 UTAUT predictors (performance expectancy, facilitating conditions, effort expectancy, and social influence). Finally, we added the score for perceived psychosocial outcomes.

Multicollinearity was assessed using variance inflation factors (VIFs). The maximum VIF among the independent variables was 3.97, with only 2 variables exceeding a value of 3. These values remain below commonly accepted thresholds, indicating that multicollinearity is not a concern.

Possibilities for creating weights to improve the match with the studied population were explored using estimates from another population survey based on a probability sample. However, the sufficient accuracy of the source estimates could not be reliably confirmed. In addition, evaluation of substantive differences between weighted and unweighted regression models, as well as a nonsignificant *F* test result [72], revealed no substantive effects of weighting on the regression estimates in any of the 3 models. We therefore used unweighted data for the analyses, while weighted regression results are reported in Multimedia Appendix 2 for comparison.

#### Data Quality and Missing Data

As noted above, respondents who did not complete at least 50% of the questionnaire were excluded from the analysis. A combination of response quality indicators was analyzed to identify potential low-quality responses, including straightlining indicators (frequency of selecting the same scale value to all items in grid questions), speeding (exceedingly short times on individual survey pages), and other response patterns (consistent tendencies to choose initial answers, midpoints, etc). No such patterns were observed among eligible respondents that would warrant further exclusions.

For the majority of statistical analyses, we used listwise deletion to maintain a consistent sample across the analyses. Of the 558 participants eligible for this study, 526 answered all items and were thus included in the regression analyses. For the reliability and factor analyses assessing measurement characteristics, we used all available data (n=558). Given the relatively low proportion of missing data, the resulting reduction in sample size is unlikely to have biased the results or meaningfully reduced statistical power.

### Ethical Considerations

The study was conducted in accordance with Slovenian and international codes of medical ethics, declarations, and conventions and with the General Data Protection Regulation [73], and it was approved by the Committee for Ethics in Research at the Faculty of Social Sciences of the University of Ljubljana on March 12, 2021 (801-2021-020/JG). This study was designed and is reported in accordance with the CHERRIES (Checklist for Reporting Results of Internet E-Surveys). The completed checklist is provided in Checklist 1.

## Results

### Characteristics of the Respondents

Table 1 presents the structure of survey respondents by key socioeconomic characteristics. They were, on average, 55.5 years old; the majority were female and in active employment. The relatively high average age and the proportion of individuals aged 70 years or older are in line with expectations, given the definition of the target population: a lower age bound of 40 years and the requirement of providing at least 4 hours of unpaid care, which becomes more likely with age.

**Table 1.** Sociodemographic characteristics, financial coping, and care burden of respondents (N=526).

Characteristic	Participants, n (%)
Sex	
Male	245 (46.6)
Female	281 (53.4)
Age (y)	
40‐49	180 (34.2)
50‐59	175 (33.3)
60‐69	109 (20.7)
70‐79	62 (11.8)
Education	
Low (ISCED^a^ 1-2)	14 (2.7)
Medium (ISCED 3-4)	271 (51.5)
High (ISCED 5+)	241 (45.8)
Employment status	
Active	328 (62.4)
Retired	140 (26.6)
Other	58 (11.0)
Financial coping	
Without problems	202 (38.4)
Just coping	244 (46.4)
Difficult or very difficult	80 (15.2)
Self-reported care burden	
Lower (0‐3)	267 (50.8)
Medium (4-6)	154 (29.3)
Higher (7-10)	105 (19.9)

a ISCED: International Standard Classification of Education.

The survey reached a low proportion of individuals with primary school education or less (International Standard Classification of Education [ISCED] 1‐2), which is likely related to the structure of the panel from which the sample was obtained.

Slightly below half of the respondents reported being able to just cope financially, while a minority (n=80, 15.2%) found their financial situation difficult or very difficult. The self-reported care burden for the majority of respondents was low, with a higher burden identified among nearly a fifth of respondents.

### Correlations Between Constructs

Behavioral intention, perceived psychosocial outcomes, performance expectancy, effort expectancy, and social influence were all positively correlated as expected (Table 2). Behavioral intention was particularly strongly correlated with perceived psychosocial outcomes and performance expectancy, whereas its correlation with facilitating conditions was relatively weak and the weakest among all correlations between behavioral intention and its predictors.

**Table 2.** Pearson correlations among Unified Theory of Acceptance and Use of Technology (UTAUT) constructs and perceived psychosocial outcomes (N=526).

Variable	BI	PPO	PE	FC	EE	SI
Behavioral intention (BI)						
*r*	1	0.68	0.66	0.23	0.32	0.49
*P* value	—^a^	<.001	<.001	<.001	<.001	<.001
Perceived psychosocial outcomes (PPO)						
*r*	0.68	1	0.83	0.11	0.36	0.47
*P* value	<.001	—	<.001	.02	<.001	<.001
Performance expectancy (PE)						
*r*	0.66	0.83	1	0.12	0.36	0.48
*P* value	<.001	<.001	—	.008	<.001	<.001
Facilitating conditions (FC)						
*r*	0.23	0.11	0.12	1	0.42	0.27
*P* value	<.001	.02	.008	—	<.001	<.001
Effort expectancy (EE)						
*r*	0.32	0.36	0.36	0.42	1	0.36
*P* value	<.001	<.001	<.001	<.001	—	<.001
Social influence (SI)						
*r*	0.49	0.47	0.48	0.27	0.36	1
*P* value	<.001	<.001	<.001	<.001	<.001	—

a Not applicable.

The correlations between the predictors varied considerably, with a strong correlation between performance expectancy and perceived psychosocial outcomes (*r*=0.83). This strong association is conceptually expected, as both constructs tap closely related evaluations of telecare’s value; performance expectancy reflects the functional usefulness of telecare, whereas perceived psychosocial outcomes capture its anticipated emotional benefits (see *Discussion*). Despite the strength of this correlation, it did not produce multicollinearity problems in the regression models: the maximum VIF among the independent variables was 3.97, which is below commonly accepted thresholds (see *Data Analysis*).

### Regression Analysis

Table 3 presents the results of 3 nested regression models. The first model contains only background variables (sociodemographics and self-reported care burden), the second model adds UTAUT predictors, and the third model adds perceived psychosocial outcomes.

**Table 3.** Nested regression models of factors associated with the behavioral intention to use telecare, including background variables, UTAUT^a^ constructs, and perceived psychosocial outcomes (N=526).

Variable	Model 1: background variables^b^			Model 2: UTAUT predictors added^c^			Model 3: perceived psychosocial outcomes added^d^		
	β^e^	*t* test (*df*)	*P* value	β	*t* test (*df*)	*P* value	β	*t* test (*df*)	*P* value
Perceived psychosocial outcomes	—^f^	—	—	—	—	—	0.41	7.19 (512)	<.001
Performance expectancy	—	—	—	0.52	13.23 (513)	<.001	0.19	3.15 (512)	.002
Facilitating conditions	—	—	—	0.11	2.80 (513)	.005	0.12	3.32 (512)	<.001
Effort expectancy	—	—	—	−0.01	−0.18 (513)	.86	−0.02	−0.60 (512)	.55
Social influence	—	—	—	0.21	5.58 (513)	<.001	0.19	5.07 (512)	<.001
Self-reported care burden	0.01	0.25 (517)	.80	−0.04	−1.31 (513)	.19	−0.02	−0.76 (512)	.45
Sex (reference: male)									
Female	−0.02	−0.40 (517)	.69	0.02	0.63 (513)	.53	0.01	0.18 (512)	.86
Age	−0.09	−1.50 (517)	.14	−0.07	−1.46 (513)	.15	−0.05	−1.19 (512)	.24
Education	0.05	1.15 (517)	.25	0.02	0.64 (513)	.52	0.04	1.12 (512)	.26
Employment status (reference: active)									
Retired	0.02	0.38 (517)	.71	0.01	0.11 (513)	.91	0.01	0.21 (512)	.83
Other	−0.05	−1.00 (517)	.32	<0.01	−0.13 (513)	.90	<0.01	0.01 (512)	.99
Financial coping (reference: no problems)									
Just coping	−0.14	−2.79 (517)	.005	−0.10	−2.55 (513)	.01	−0.09	−2.43 (512)	.02
Difficult or very difficult	−0.06	−1.22 (517)	.22	−0.05	−1.28 (513)	.20	−0.04	−1.07 (512)	.29

a UTAUT: Unified Theory of Acceptance and Use of Technology.

b Model 1: *F* test (residual *df*)=2.07 (517), *P*=.04, *R*² (coefficient of determination)=0.03, adjusted *R*²=0.02, Akaike information criterion=1236.30, Bayesian information criterion=1274.69.

c Model 2: *F* test (residual *df*)=41.06 (513), *P*<.001, *R*² (coefficient of determination)=0.49, adjusted *R*²=0.48, *R*² change from Model 1=0.46; likelihood ratio test vs Model 1, *P*<.001, Akaike information criterion=906.80, Bayesian information criterion=962.25.

d Model 3: *F* test (residual *df*)=45.62 (512), *P*<.001, *R*² (coefficient of determination)=0.54, adjusted *R*²=0.52, *R*² change from Model 2=0.05; likelihood ratio test vs Model 2, *P*<.001, Akaike information criterion=858.18, Bayesian information criterion=917.89.

e Standardized regression coefficient.

f Not applicable.

The model containing only background variables explains behavioral intention relatively poorly, with financial coping being the only significant predictor. The proportion of explained variance is low (3%), although the overall model fit is significant. The inclusion of UTAUT predictors significantly improves the model fit and its explanatory power, increasing the proportion of explained variance by 46 percentage points. The associations of performance expectancy, social influence, and facilitating conditions with behavioral intention are significant and positive. Consistent with the initial model, those struggling to cope financially are less willing to invest time and money.

The addition of perceived psychosocial outcomes again significantly improves model fit and also increases the explained variance. While this increase may seem modest, the added variable has the strongest effect size among all predictors, with a positive effect as expected. The pattern of observed relationships between the other predictors and the dependent variable is consistent with the previous model, except for performance expectancy, which has a markedly smaller effect size. The 2 constructs share a high proportion of the explained variance. Perceived psychosocial outcomes are associated with a reduction in the effect size of performance expectancy.

## Discussion

### Principal Findings and Comparison With Prior Work

In this study, perceived psychosocial outcomes emerged as the strongest predictor of informal caregivers’ behavioral intention to use telecare, and adding these outcomes significantly improved the explanatory power of the UTAUT model. Performance expectancy, social influence, and facilitating conditions were also significant predictors, whereas effort expectancy and self-reported care burden were not.

This study extended the UTAUT model by incorporating perceived psychosocial outcomes to explain the behavioral intention of Slovenian informal caregivers of older adults to use telecare. While existing acceptance models provide a useful foundation, they do not consider psychosocial factors that are central to caregiving contexts [23,44-46]. To address this gap, we investigated whether including perceived psychosocial outcomes (such as reduced anxiety and increased peace of mind) improves the UTAUT model’s explanatory power beyond conventional sociodemographic variables and standard UTAUT predictors (performance expectancy, effort expectancy, social influence, and facilitating conditions).

The model including only background variables showed limited explanatory power, which is consistent with previous research showing mixed or weak effects of sociodemographic factors on behavioral intention [74-76]. Financial coping (ie, having sufficient income) was the only statistically significant sociodemographic variable associated with behavioral intention. This finding aligns with previous studies in which coping financially was found to be an important factor in informal caregivers’ acceptability of telecare [9,75-77]. Self-reported caregiving burden was not a significant predictor, although prior research has indicated that higher burden may increase the need for support [25,47,78]. One possible explanation is that, before caregivers have actually used telecare, their intention to do so may depend more on what they expect it to offer (its practical and psychosocial benefits) than on how much strain they are currently under. Such strain might instead translate into actual uptake only once the technology is available and its relevance to a specific caregiving situation becomes apparent. The single-item measurement of subjective burden may also have limited its predictive value, a point to which we return in the *Strengths and Limitations* section.

Adding the UTAUT factors significantly improved the model’s explanatory power. Performance expectancy, social influence, and facilitating conditions were significant positive predictors, in line with previous research [23,25,56,79]. However, the relatively weak effect of facilitating conditions suggests that in the preadoption stage, structural and resource-related aspects may play a less prominent role compared with performance expectancy and social influence. Effort expectancy was not a significant predictor, which is likely due to the respondents’ lack of direct experience with telecare [80]. Without hands-on experience, users may find it difficult to assess ease of use [23,80,81], whereas evidence identifying effort expectancy as a significant predictor largely comes from postimplementation research involving participants with direct experience of the technology [82-84]. This suggests that cross-sectional studies (especially those involving nonusers) may need to consider alternative or additional constructs. For instance, assessing caregivers’ perceptions of the importance of ease of use may be more germane than asking them to estimate the ease of use of hypothetical or unfamiliar technologies.

Although UTAUT is a robust model for predicting behavioral intention to use telecare, our results show that extending it with perceived psychosocial outcomes is well justified. Their inclusion in the UTAUT model improved its explanatory power. Psychosocial outcomes showed the strongest association among all variables, indicating their key role in explaining informal caregivers’ preuse behavioral intention to use telecare. This inclusion substantially reduced the traditional dominance of performance expectancy in explaining telecare acceptability. This may suggest that psychosocial outcomes partially mediate the relationship between performance expectancy and behavioral intention. However, given the study design, mediation cannot be formally established. An alternative interpretation is that psychosocial outcomes represent a distinct but closely related dimension of perceived usefulness that is not fully captured by standard measures of performance expectancy. In line with the original formulation of the technology acceptance model and its extensions, perceived usefulness (and performance expectancy) is typically defined in terms of functional and performance-related benefits, such as efficiency and effectiveness [40]. At the same time, research in telecare and caregiving contexts shows that emotional benefits, such as reassurance, peace of mind, and reduced anxiety, are central to how technologies are evaluated and are often treated as either implicit components of usefulness or as distinct experiential drivers of acceptability [56,85,86]. Notably, while quantitative models tend to emphasize functional utility as the primary predictor of acceptance, qualitative research consistently identifies emotional benefits as key motivations underlying caregivers’ decisions [24,25,47,55], suggesting that these dimensions may be insufficiently captured in standard model operationalizations. The strong association between performance expectancy and psychosocial outcomes observed in this study is consistent with this partial conceptual overlap, with performance expectancy reflecting functional usefulness and psychosocial outcomes capturing anticipated emotional benefits, while their independent effects indicate that psychosocial outcomes contribute additional explanatory value. These findings indicate that psychosocial outcomes represent a central, yet still underrepresented, dimension of telecare acceptability in existing technology acceptance models.

### Implications for Practice, Policy, and Future Research

These findings have practical implications for how telecare solutions are designed, implemented, and supported in policy. In addition to functional features, telecare services should address the psychosocial needs of informal caregivers by providing reassurance, reducing anxiety, and enhancing a sense of safety. These aspects are often overlooked in favor of technical features or the needs of care recipients, despite informal caregivers often codeciding on the use of telecare [22]. Developers, service providers, and policymakers should therefore consider these dimensions when designing, communicating, implementing, and evaluating telecare solutions.

Based on our findings, future research should look at how the influence of psychosocial outcomes varies at different stages of the acceptance process. While our study focused on the initial stage of the user journey (ie, preuse acceptability), actual use of the technology and user engagement are likely to shape acceptance during both the initial and sustained use stages [43]. Consequently, the predictive strengths of specific acceptance factors, including psychosocial outcomes, may vary at these stages. Future studies could also further examine the interaction between functional and psychosocial determinants of telecare acceptance, including by testing whether psychosocial outcomes mediate the relationship between performance expectancy and behavioral intention and how these relationships evolve over time. In addition, there is a need to better integrate insights from qualitative research into quantitative models, particularly through the development of more refined measures of psychosocial outcomes.

### Strengths and Limitations

A strength of this study is its focus on the theoretical framework for the acceptability of telecare by informal caregivers of older adults, which is often overlooked. The study also introduces a new dimension, namely the psychosocial impact of telecare use, which existing empirical models of telecare acceptability have largely neglected [23,43].

However, it has several limitations. As a cross-sectional survey of self-reported intention, the study describes associations rather than causal relationships, and behavioral intention at the preuse stage is not equivalent to actual telecare use.

First, the sampling relied on a nonprobability online panel due to the absence of a dedicated sampling frame for informal caregivers in Slovenia. This may introduce self-selection bias and limit the representativeness of the results, as individuals who participate in online panels may differ systematically from the broader population of informal caregivers. The lack of a reliable source of population-level data on caregivers in Slovenia limits our ability to assess how representative the sample is of the broader caregiver population. A comparison with a similar target group in an independent, probability-based population survey suggests that the panel sample somewhat overrepresents female (by 3.3 percentage points), highly educated (by 8.3 percentage points), and younger respondents (2.2 y younger on average), with urban and employed respondents also likely overrepresented. Because the 2 surveys differed in question design and coverage, a formal statistical comparison of sample structures was not possible, and these findings should be interpreted as indicative. In addition, respondents with greater familiarity with digital environments may be overrepresented, making the results less representative for less digitally engaged caregiver populations. At the same time, as telecare services typically require access to and basic proficiency with digital technologies, such as a smartphone and an internet connection, individuals without such access would be unlikely to use these services even if they were available. To mitigate this problem, we applied poststratification weights based on data from the representative Slovenian Public Opinion Survey [64], which improved the accuracy of population estimates. However, regression analyses were conducted on unweighted data, as weighting did not substantively affect the results. The findings should therefore be interpreted with caution when generalizing to caregiver populations that are less digitally engaged or to contexts outside Slovenia.

Second, as with all survey-based research, the survey instrument and context may have influenced the results. In particular, social desirability bias may affect responses, especially when reporting perceived psychosocial outcomes, which may result in more favorable assessments. Although self-administration and the assurance of confidentiality generally reduce such effects, their influence cannot be entirely excluded. Furthermore, because all variables were collected using the same survey instrument, the observed relationships may be partially influenced by common method variance, which could inflate correlations between constructs. However, the Harman single-factor test did not indicate that this is a major concern. In addition, caregiving burden was measured using a single-item indicator, which, although commonly applied, may not adequately reflect the multidimensional nature of caregiving experiences [66]. The facilitating conditions scale also showed limited, albeit acceptable, reliability, which indicates the need to improve the measurement characteristics of the scale.

Third, the study surveyed family caregivers aged 40 years and older because international research suggests caregiving prevalence peaks in middle adulthood and remains high into older age [87,88]. This decision is further supported by data from the Slovenian Public Opinion Survey [64], indicating that 77% of informal caregivers of older adults fall within this age range.

Fourth, contrary to expectations, care burden was not a significant factor in our model, which may be due to the use of a single-item subjective burden measure that may not adequately account for the complexity of caregiving experiences. Additionally, self-reported measures are prone to biases, such as social desirability, caregiver guilt, and individual coping mechanisms. Perceptions of burden may also fluctuate over time, further affecting the accuracy of the assessment. Future studies should therefore incorporate both subjective and objective measures of care burden to ensure a more complete assessment.

Fifth, the operationalization of behavioral intention also as willingness to pay for telecare services may introduce bias related to respondents’ financial situation, as individuals with fewer financial resources may express lower intention independent of their attitudes toward the technology. To partially account for this, financial coping was included as a control variable in the regression models, although it may not capture all income-related influences on the measure. Relatedly, the 2 items used to operationalize behavioral intention capture conceptually distinct facets of motivational readiness: willingness to invest time reflects an attitudinal commitment to engaging with the technology, whereas willingness to pay reflects an economic commitment that is more strongly conditioned by personal financial circumstances. Treating them as a single measure is therefore a simplification, since it may blur the line between the attitudinal and economic sides of intention. Future research should measure attitudinal intention and economic willingness separately to better untangle the role each one plays in the acceptability of telecare.

Finally, this study was conducted in a single country, and the relatively low representation of participants with lower educational levels may further limit the generalizability of the findings, as this group may have different levels of digital literacy and attitudes toward telecare. Differences in informal care systems, elder care practices, and the availability of telecare solutions may also limit the transferability of our findings to other contexts.

Despite these limitations, our findings offer a better understanding of informal caregivers’ acceptability of telecare. They may help research and policy across different countries and for different technologies.

### Conclusions

This study aimed to assess telecare acceptability among informal caregivers of older people and to identify relevant acceptance factors. Our findings show that adding perceived psychosocial outcomes of telecare engagement significantly improved the explanatory power of the UTAUT model and that these outcomes were the predictor most strongly associated with self-reported behavioral intention. This suggests that psychosocial factors are key to telecare acceptability within informal caregiving contexts. Their inclusion goes beyond improving the model empirically by also advancing it theoretically, as it incorporates the emotional aspects of caregiving (peace of mind, reduced anxiety, sense of safety, and perceived connectedness with the care recipient) that traditional technology acceptance models do not capture. Developers, policymakers, and health care providers should consider psychosocial outcomes when designing, testing, and deploying telecare solutions, as addressing informal caregivers’ needs for reassurance, safety, and reduced anxiety may be important for their adoption. This can be translated into practice by incorporating caregiver perspectives into user-centered design, addressing psychosocial outcomes in implementation, and reflecting them in evaluation processes. Future research should examine how psychosocial outcomes influence technology acceptance at different stages, such as preadoption, initial adoption, and sustained use, to better understand how this influence changes over time.

## Supplementary material

10.2196/85534 Multimedia Appendix 1 Variables (constructs) and corresponding questionnaire items used in regression models.

10.2196/85534 Multimedia Appendix 2 Comparison of regression models of factors associated with behavioral intention to use telecare based on weighted and unweighted data.

10.2196/85534 Checklist 1 CHERRIES checklist.

## Abbreviations

CFI: comparative fit index
ISCED: International Standard Classification of Education
RMSEA: root mean square error of approximation
SRMR: standardized root mean square residual
TLI: Tucker-Lewis index
UTAUT: Unified Theory of Acceptance and Use of Technology
VIF: variance inflation factor
