# Supplementary material for: Integrating Perceived Psychosocial Outcomes Into the Assessment of Telecare Acceptability Among Slovenian Informal Caregivers of Older People: Cross-Sectional Survey Study
Source: JMIR Aging. 2026 Oct 20;9:e85534. doi: 10.2196/85534 (PMC13684353; doi:10.2196/85534)
Supplement: Supplementary file 1 [file aging-v9-e85534-s003.pdf]

Checklist for Reporting Results of Internet E-Surveys (CHERRIES).

Item category	Checklist item	Explanation
**Design**	Description of survey design	Cross-sectional closed web survey of a nonprobability online access panel. Target population: Slovenian residents aged 40 years or older who had provided at least 4 hours of unpaid care per week to a family member or relative aged 65 years or older during the previous 6 months. The sample was drawn from the panel by quota sampling defined by statistical region (NUTS-3), gender, and 10-year age group to approximate the structure of the general population.
**IRB approval and informed consent process**	IRB approval	The study was approved by the Committee for Ethics in Research at the Faculty of Social Sciences of the University of Ljubljana on 12 March 2021 (801-2021-020/JG) and conducted in accordance with Slovenian and international codes of medical ethics and the General Data Protection Regulation.
	Informed consent	The survey was administered to members of an established online access panel. Before starting the questionnaire, respondents were informed about the voluntary nature of participation, the length of the survey, and the data protection measures. The panel provider adheres to the ESOMAR International Code, under which respondents decide freely on participation, may withdraw at any time, and may refuse to answer any question.
	Data protection	No personally identifiable information about the participants was obtained by the research team. Panel membership data were held by the panel provider and were not linked to the survey responses available to the researchers.
**Development and pre-testing**	Development and testing	The questionnaire was developed by the research team on the basis of the theoretical model and a systematic review of measurement instruments. Instruments without an existing Slovenian version were translated following the TRAPD approach (Translation, Review, Adjudication, Pretesting, Documentation). The questionnaire was evaluated in a multistage process between April 2020 and April 2021: initial pilot testing with informal caregivers (n=93), testing by the research team and cognitive interviews (n=9), an expert methodological review (n=4; January 2021), and a final pilot survey administered through the online panel in April 2021, in which 49 eligible caregivers completed the questionnaire.
**Recruitment process and description of the sample having access**	Open survey versus closed survey	Closed survey. Access was limited to invited panel members through unique access codes; each respondent could participate only once.
	Contact mode	Panel members were invited by e-mail by the panel provider. At least one e-mail reminder was sent to nonrespondents

**to the questionnaire**		before additional panelists were contacted to meet the preset quotas.
	Advertising the survey	The survey was not advertised publicly. Invitations were sent only to panel members selected by quota sampling.
**Survey administration**	Web/E-mail	The survey was administered as a web questionnaire; invitations and reminders were sent by e-mail.
	Context	The survey was administered within a Slovenian commercial online access panel, selected after assessment against the ESOMAR guidelines for online panels. Eligibility was established with a screening web questionnaire (age, types of care provided, average weekly hours of care); eligible respondents were routed immediately to the main questionnaire.
	Mandatory/voluntary	Participation was voluntary.
	Incentives	Respondents who completed the questionnaire were assigned value points in accordance with the standard incentivization practice of the panel provider.
	Time/Date	Data were collected in two rounds: a pilot round from 9 to 13 April 2021 and the main round from 19 April to 6 May 2021. Both rounds used the same main questionnaire and are included in the dataset.
	Randomization of items or questionnaires	Randomization was applied selectively. For some multi-item instruments, the order of items within grid questions was randomized; for others, the original item order was retained. The order of question blocks was not randomized.
	Adaptive questioning	Adaptive questioning was used: inapplicable questions were automatically skipped on the basis of relevant previous answers, and the screening questionnaire routed only eligible respondents to the main questionnaire.
	Number of Items	The main questionnaire (excluding the screening questionnaire) comprised questions numbered Q1 to Q64, several consisting of multiple items (133 variables in total). Questions were presented one per screen, with grid questions split across two or more screens (see Number of screens below). The number of questions presented varied between respondents because of adaptive questioning. The mean completion time of the main questionnaire was 17 minutes.
	Number of screens (pages)	The questionnaire was presented with one question per screen. Grid questions comprising a larger number of items were split across two or more screens. The exact total number of screens was not recorded and varied between respondents because of adaptive questioning.
	Completeness check	Responses to individual questions were not forced: respondents were allowed to skip individual questions.

		Completeness was addressed at the analysis stage (see Analysis below).
	Review step	Respondents were able to go back and change their answers until the questionnaire was completed.
**Response rates**	Unique site visitor	Because this was a closed survey, unique visitors correspond to the invited panelists: 3284 panel members received an e-mail invitation, of whom 1974 (60.1%) opened the survey link.
	View rate (Ratio of unique survey visitors/unique site visitors)	Of the 3284 invited panelists, 1974 (60.1%) opened the survey link; 1310 (39.9%) never clicked the link.
	Participation rate (Ratio of unique visitors who agreed to participate/unique first survey page visitors)	Of the 1974 panelists who opened the survey link, 1834 (92.9%) completed the screening questionnaire (140 opened the link without completing the screening). Of these, 781 met the eligibility criteria of the screening questionnaire and entered the main questionnaire (23.8% of all invited panelists).
	Completion rate (Ratio of users who finished the survey/users who agreed to participate)	Of the 781 eligible respondents entering the main questionnaire, 742 valid unique responses remained after removing 2 entries with an invalid unique identifier and 37 duplicate entries; a further 2 straightlining responses were removed, and 637 respondents (85.8% of the 742 valid unique responses; 19.4% of all invited panelists, following AAPOR standards) completed at least 50% of the main questionnaire. Contextual eligibility screening then excluded 2 respondents who did not provide at least 4 hours of care per week and 23 whose care recipient was younger than 65 years, yielding the final dataset of 612 respondents (18.6% of invited panelists). Of these, 558 were retained for analysis after excluding current or former users of care-related assistive technologies (see Analysis below).
**Preventing multiple entries from the same individual**	Cookies used	The survey was accessible only through personalized invitation links with unique access codes, and each respondent could participate only once. Duplicate entries were additionally identified and removed at the analysis stage on the basis of the unique respondent identifier. Whether the survey platform additionally set cookies to identify client computers was not recorded.
	IP check	Duplicate entries were identified and removed at the analysis stage on the basis of the unique respondent identifier; 37 duplicate entries were excluded. In addition, the panel provider processes an online identifier and web browser properties to prevent fraud and data falsification at the data collection stage.
	Log file analysis	Paradata were analyzed at the analysis stage: entries with an invalid unique identifier (n=2) and duplicate entries (n=37) were removed, straightlining was assessed from the standard deviation of responses to grid questions (2

		responses excluded), and response times on individual survey pages were examined to identify speeding. No further log file analysis was performed.
	Registration	The survey was closed: access was limited to invited panel members through personalized invitation links with unique access codes, which were intended to permit a single response per panelist. The pilot and main data collection rounds were subsequently merged into a single dataset on the basis of the respondent identifier, and 37 records with a duplicated identifier and 2 records with an invalid identifier were detected and removed before analysis, leaving 742 valid unique responses.
**Analysis**	Handling of incomplete questionnaires	Respondents who completed less than 50% of the main questionnaire items were excluded (see Completion rate above). For the analyses, listwise deletion was applied in the regression models (n=526 with complete data on all model variables), while correlational and factor analyses used all available data (n=558). Item nonresponse was low because respondents were allowed, but not forced, to answer every question.
	Questionnaires submitted with an atypical timestamp	Response quality indicators were analyzed to identify potential low-quality responses, including speeding (exceedingly short times on individual survey pages), straightlining (assessed from the standard deviation of responses to grid questions), and other response patterns (consistent tendency to choose initial answers or midpoints). Two straightlining responses were excluded during technical data cleaning; no further patterns warranting exclusion were observed among eligible respondents.
	Statistical correction	The feasibility of weighting was explored. Because no population data on informal caregivers exist in Slovenia, the screening questions were embedded in a probability-based national survey (Slovenian Public Opinion Survey; n=1022, of whom 225 met the same eligibility criteria) to approximate the sociodemographic structure of the caregiver population, and raking weights (gender by age group, region by settlement type, calibrated against national census data) were derived from it. Because the accuracy of the source estimates could not be reliably confirmed and weighting had no substantive effect on the regression estimates, unweighted data were used for the analyses; weighted regression results are reported in Multimedia Appendix 2 for comparison.
