# Supplementary material for: Integrating Perceived Psychosocial Outcomes Into the Assessment of Telecare Acceptability Among Slovenian Informal Caregivers of Older People: Cross-Sectional Survey Study
Source: JMIR Aging. 2026 Oct 20;9:e85534. doi: 10.2196/85534 (PMC13684353; doi:10.2196/85534)
Supplement: Supplementary file 2 [file aging-v9-e85534-s001.docx]

Variables (constructs) and corresponding questionnaire items used in regression models.

| **Variable (construct)** | **Questionnaire items and response options^a^** |
| --- | --- |
| Behavioral intention | I would be willing to devote some time to learn how to properly use services.  I would be willing to pay 25 EUR per month for services.  (1: completely disagree ... 5: completely agree) |
| Perceived psychosocial outcomes | I would feel more at ease.  I would feel less worried about the person’s condition.  I would feel that the person I am helping is safe.  I would feel more connected to the person I am helping.  (1: completely disagree ... 5: completely agree) |
| Performance expectancy | It would save me time.  It would make it easier for me to assist the person.  It would make it easier for me to carry out my other daily tasks.  These services would improve my quality of life.  Overall, I think these services would be useful in my life.  (1: completely disagree ... 5: completely agree) |
| Facilitating conditions | I have enough money to use these services.  I have the knowledge necessary to use these services.  I know someone who would be available for assistance with such services if I needed it.  (1: completely disagree ... 5: completely agree) |
| Effort expectancy | Overall, I would find these services easy to use.  (1: completely disagree ... 5: completely agree) |
| Social influence | If health or other professional staff (e.g., home caregivers) suggested that the person I am helping should use these services, they would probably use them.  The person I am assisting would resist accepting these services.  (1: completely disagree ... 5: completely agree) |
| Self-reported care burden | On a scale from 0 to 10, how burdensome is it for you to provide care for this person?  (0: providing care is not burdensome at all ...  10: providing care is extremely burdensome) |
| Gender | Gender (male/female) |
| Age | Year of birth (age calculated) |
| Education | Highest completed education level (11 education levels of the Slovenian schooling system) |
| Employment status | How would you describe your current employment status?  (Retired without additional employment / Retired with additional employment / Employed / Self-employed / Farmer / Homemaker / Unemployed / Permanently unable to work / Other: [text field]) |
| Financial coping | How do you view your household’s income situation? Which of the following describes it best?  (With our current income, we can live easily without difficulty / With our current income, we just manage / With our current income, we find it hard to get by /  With our current income, we find it extremely hard to get by) |

^a^Translated from the Slovenian text used in the questionnaire.
