# Supplementary material for: Integrating Perceived Psychosocial Outcomes Into the Assessment of Telecare Acceptability Among Slovenian Informal Caregivers of Older People: Cross-Sectional Survey Study
Source: JMIR Aging. 2026 Oct 20;9:e85534. doi: 10.2196/85534 (PMC13684353; doi:10.2196/85534)
Supplement: Supplementary file 3 [file aging-v9-e85534-s002.docx]

Comparison of regression models of factors associated with behavioral intention to use telecare based on weighted and unweighted data.

**Model based on unweighted data**

|  | Model 1: Background variables | | | Model 2: UTAUT^a^ predictors added | | | Model 3: Perceived psychosocial outcomes added | | |
| --- | --- | --- | --- | --- | --- | --- | --- | --- | --- |
|  | *b*^b^ | *t* | *P* | *b* | *t* | *P* | *b* | *t* | *P* |
| Perceived psychosocial outcomes | —^c^ | — | — | — | — | — | 0.42 | 7.19 | <.001 |
| Performance expectancy | — | — | — | 0.55 | 13.23 | <.001 | 0.20 | 3.15 | .002 |
| Facilitating conditions | — | — | — | 0.11 | 2.80 | .005 | 0.12 | 3.32 | <.001 |
| Effort expectancy | — | — | — | -0.01 | -0.18 | .86 | -0.02 | -0.60 | .55 |
| Social influence | — | — | — | 0.24 | 5.58 | <.001 | 0.21 | 5.07 | <.001 |
| Self-reported care burden | 0.00^d^ | 0.25 | .80 | -0.01 | -1.31 | .19 | -0.01 | -0.76 | .45 |
| Sex (*ref*. male) |  |  |  |  |  |  |  |  |  |
| Female | -0.03 | -0.40 | .69 | 0.03 | 0.63 | .53 | 0.01 | 0.18 | .86 |
| Age | -0.00^d^ | -1.50 | .14 | -0.01 | -1.46 | .15 | -0.00^d^ | -1.19 | .24 |
| Education | 0.04 | 1.15 | .25 | 0.01 | 0.64 | .52 | 0.02 | 1.12 | .26 |
| Employment status (*ref*. active) |  |  |  |  |  |  |  |  |  |
| Retired | 0.04 | 0.38 | .71 | 0.01 | 0.11 | .91 | 0.02 | 0.21 | .83 |
| Other | -0.12 | -1.00 | .32 | -0.01 | -0.13 | .90 | 0.00^d^ | 0.01 | .99 |
| Financial coping (*ref*. no problems) |  |  |  |  |  |  |  |  |  |
| Just coping | -0.21 | -2.79 | .005 | -0.15 | -2.55 | .01 | -0.14 | -2.43 | .02 |
| Difficult or very difficult | -0.14 | -1.22 | .22 | -0.11 | -1.28 | .20 | -0.09 | -1.07 | .29 |
| **Model summary** | | | | | | | | | |
| *F* test (residual df) | 2.07 (517) | | | 41.06 (513) | | | 45.62 (512) | | |
| *P* | .04 | | | <.001 | | | <.001 | | |
| *R*^2e^ | 0.03 | | | 0.49 | | | 0.54 | | |

^a^UTAUT: Unified Theory of Acceptance and Use of Technology.

^b^Unstandardized regression coefficient.

^c^Not applicable.

^d^Absolute value of the coefficient is smaller than 0.01.

^e^Coefficient of determination.

**Model based on weighted data**

|  | Model 1: Background variables | | | Model 2: UTAUT^a^ predictors added | | | Model 3: Perceived psychosocial outcomes added | | |
| --- | --- | --- | --- | --- | --- | --- | --- | --- | --- |
|  | *b*^b^ | *t* | *P* | *b* | *t* | *P* | *b* | *t* | *P* |
| Perceived psychosocial outcomes | —^c^ | — | — | — | — | — | 0.46 | 6.32 | <.001 |
| Performance expectancy | — | — | — | 0.56 | 10.25 | <.001 | 0.17 | 2.32 | .02 |
| Facilitating conditions | — | — | — | 0.12 | 2.69 | .007 | 0.14 | 3.49 | <.001 |
| Effort expectancy | — | — | — | -0.01 | -0.25 | .81 | -0.02 | -0.46 | .65 |
| Social influence | — | — | — | 0.22 | 3.75 | <.001 | 0.19 | 3.38 | <.001 |
| Self-reported care burden | 0.00^d^ | 0.31 | .76 | -0.01 | -1.00 | .32 | -0.00^d^ | -0.34 | .74 |
| Sex (*ref*. male) |  |  |  |  |  |  |  |  |  |
| Female | 0.01 | 0.18 | .86 | 0.08 | 1.38 | .17 | 0.05 | 1.06 | .29 |
| Age | -0.01 | -1.28 | .20 | -0.01 | -1.25 | .21 | -0.00^d^ | -0.95 | .34 |
| Education | 0.01 | 0.61 | .55 | -0.01 | -0.06 | .95 | 0.01 | 0.44 | .66 |
| Employment status (*ref*. active) |  |  |  |  |  |  |  |  |  |
| Retired | 0.09 | 0.70 | .49 | 0.01 | 0.17 | .87 | 0.02 | 0.26 | .79 |
| Other | -0.07 | -0.64 | .52 | -0.05 | -0.53 | .59 | -0.04 | -0.42 | .67 |
| Financial coping (*ref*. no problems) |  |  |  |  |  |  |  |  |  |
| Just coping | -0.21 | -2.68 | .008 | -0.18 | -2.81 | .005 | -0.16 | -2.82 | .005 |
| Difficult or very difficult | -0.14 | -1.33 | .18 | -0.11 | -1.26 | .21 | -0.07 | -0.85 | .40 |
| **Model summary** | | | | | | | | | |
| *F* test (residual df) | 1.43 (517) | | | 35.56 (513) | | | 41.50 (512) | | |
| *P* | .18 | | | <.001 | | | <.001 | | |
| *R*^2e^ | 0.02 | | | 0.50 | | | 0.55 | | |

^a^UTAUT: Unified Theory of Acceptance and Use of Technology.

^b^Unstandardized regression coefficient.

^c^Not applicable.

^d^Absolute value of the coefficient is smaller than 0.01.

^e^Coefficient of determination.
